# Peroxisome proliferator-activated receptor-gamma dependent pathway reduces the phosphorylation of dynamin-related protein 1 and ameliorates hippocampal injury induced by global ischemia in rats

**DOI:** 10.1186/s12929-016-0262-3

**Published:** 2016-05-12

**Authors:** Yao-Chung Chuang, Tsu-Kung Lin, Ding-I Yang, Jenq-Lin Yang, Chia-Wei Liou, Shang-Der Chen

**Affiliations:** Department of Neurology, Kaohsiung Chang Gung Memorial Hospital, Chang Gung University College of Medicine, Kaohsiung, Taiwan; Center for Translational Research in Biomedical Sciences, Kaohsiung Chang Gung Memorial Hospital, Kaohsiung, Taiwan; Department of Neurology, Faculty of Medicine, College of Medicine, Kaohsiung Medical University, Kaohsiung, Taiwan; Institute of Brain Science, National Yang-Ming University, Taipei, Taiwan

**Keywords:** Apoptosis, Dynamin-related protein 1, Global ischemia, Hippocampus, Peroxisome proliferator-activated receptor-gamma, Pioglitazone

## Abstract

**Background:**

Dynamin-related protein 1 (Drp1) is a mitochondrial fission protein that, upon phosphorylation at serine 616 (p-Drp1(Ser616)), plays a pivotal role in neuronal death after ischemia. In the present study, we hypothesized that peroxisome proliferator-activated receptor-gamma (PPARγ)-dependent pathway can reduce the expression of p-Drp1(Ser616) and ameliorate hippocampal injury induced by global ischemia in rats.

**Results:**

We found that pretreatment of the rats with Mdivi-1, a selective Drp1 inhibitor, decreased the level of transient global ischemia (TGI)-induced p-Drp1(Ser616) and reduced cellular contents of oxidized proteins, activated caspase-3 expression as well as the extent of DNA fragmentation. Delivery of siRNA against Drp1 attenuated the expression of p-Drp1(Ser616) that was accompanied by alleviation of the TGI-induced protein oxidation, activated caspase-3 expression and DNA fragmentation in hippocampal proteins. Exogenous application of pioglitazone, a PPARγ agonist, reduced the p-Drp1(Ser616) expression, decreased TGI-induced oxidative stress and activated caspase-3 expression, lessened the extents of DNA fragmentation, and diminished the numbers of TUNEL-positive neuronal cells; all of these effects were reversed by GW9662, a PPARγ antagonist.

**Conclusions:**

Our findings thus indicated that inhibition of TGI-induced p-Drp1(Ser616) expression by Drp1 inhibitor and Drp1-siRNA can decrease protein oxidation, activated caspase-3 expression and neuronal damage in the hippocampal CA1 subfield. PPARγ agonist, through PPARγ-dependent mechanism and via decreasing p-Drp1(Ser616) expression, can exert anti-oxidative and anti-apoptotic effects against ischemic neuronal injury.

## Background

Mitochondria are the powerhouses of cells to produce ATP as well as to regulate signaling cascades, including apoptosis [1]. A recent progress towards the understanding of mitochondrial control over apoptosis is the discovery of a drastic morphological change of this organelle under stressful conditions [2, 3]. Mitochondria are dynamic organelles that maintain their shape or morphology via two opposing processes: fission and fusion [4–6]. While the fission process involves the constriction and cleavage of mitochondria, fusion process involves the lengthening of mitochondria by tethering and joining two adjacent mitochondria together [4–6]. It was shown that, just before the apoptotic processes, mitochondria fragment into multiple small units (fission) and blocking mitochondrial fission can inhibit cytochrome *c* release with delayed cell death [2]. Drp1, an important fission protein, plays a crucial role in focal cerebral ischemia and inhibition of Drp1 can reduce the infarct volumes [7–9]. Expression of the dominant-negative Drp1 mutant in cell lines decreases mitochondrial fragmentation and blocks cell death in response to various apoptotic insults [10, 11].

A selective neuronal loss in hippocampal CA1 subfield is a histological hallmark of transient global ischemia (TGI) and reperfusion [12, 13]. This condition occurs in patients with anoxic-ischemic encephalopathy and cardiorespiratory arrest of various causes [14] The damage may cause long-term cognitive change as the hippocampus is an important player in memory consolidation and in conjunction with other cortical areas, is critical for the retrieval of remote episodic memories and anterograde memory processes [15]. Emerging evidence suggests that the mitochondrion may play a major role in delayed neuronal death of the CA1 subfield after TGI [16–19]. Recent evidence implicates the regulatory processes of mitochondrial biogenesis as a protective effect in the pathogenesis of cerebral ischemia [18, 20]. Lately, mitochondrial dynamic has been recognized as a pivotal process in regulating cell survival and death; in particular, mitochondrial fission occurs as an upstream and early event in neuronal death after cerebral ischemia [7–9]. Recently, we have also shown that TGI induced a transient increase in the phosphorylation of Drp1 at serine 616 (p-Drp1(Ser616)), without significantly affecting the expression of total Drp1 proteins or its phosphorylation at serine 637, in the rat hippocampal CA1 proteins [21]. Together these findings support the crucial roles of mitochondrial dynamics in ischemic neuronal death.

Peroxisome proliferator-activated receptor gamma (PPARγ) agonist, such as pioglitazone or rosiglitazone has been shown to reduce inflammation [22, 23], decrease oxidative damage [23–27], and reduce cell death following ischemic injury. Despite its ability of enhancing mitochondrial biogenesis [28], however, the potential correlation between PPARγ agonist and mitochondrial dynamics, in particular its effects on the expression of p-Drp1(Ser616), has never been investigated before. To resolve this question, we hypothesized that PPARγ-dependent mechanism may exert anti-oxidative and anti-apoptotic effects against TGI in rats which involves p-Drp1(Ser616) expression. Results derived from this work may further improve our understandings towards the molecular mechanisms underlying TGI-induced neuronal demise in the hippocampal CA1 subfield and provide novel insights for future development of therapeutic regimen.

## Methods

### Animals and general preparations

All the animal studies followed the Guide for the Care and Use of Laboratory Animals, National Research Council, USA and were approved by the Institutional Animal Care and Use Committee (IACUC) of Chang Gung Memorial Hospital (Permit Number: 2009121704). All efforts were made to minimize animal suffering and to reduce the number of animals sacrificed. Adult male Sprague-Dawley rats (250–325 g) were purchased from BioLASCO, Taiwan. They were housed in an Association for Assessment and Accreditation of Laboratory Animal Care (AAALAC), an internationally accredited animal facility, under temperature control (24–25 °C) and 12-h light-dark cycle. Standard laboratory rat chow and tap water were available ad libitum. Animals were anesthetized with chloral hydrate (400 mg/kg, i.p.) to perform preparative surgery. An experimental model of TGI was performed as previously reported [29] with modifications [19, 21]. Briefly, the animals were subjected to a 10-min period of global ischemia by clamping both common carotid arteries and lowering blood pressure to 35–40 mm-Hg by withdrawing blood from a femoral arterial catheter; blood pressure was restored by infusing the withdrawn blood afterwards. A PE-50 catheter was inserted to femoral artery to continuously record the arterial blood pressure and to keep blood pressure within 35–40 mm-Hg. After regaining consciousness, the animals were maintained in an air-conditioned room at 25 °C.

### Pharmacological pretreatments

In the experiments involving pharmacological pretreatments, the dosage for Mdivi-1 was tested as previously reported [8, 30], the dosage of GW9662 and pioglitazone were based on our previous studies [26, 31]. One group of rats were treated intraperitoneally with Drp1 inhibitor Mdivi-1 (2.4 mg/kg), which was purchased from Sigma-Aldrich Ltd (St. Louis, MO, USA), or the solvent dimethyl sulfoxide (DMSO) 30 min before TGI. The other group of rats were microinjected into bilateral CA1 subfields with pioglitazone (Cayman Chemical, Ann Arbor, MI, USA; 20 nmol), GW9662 (Cayman Chemical, 500 ng) or DMSO as the vehicle and volume control 30 min before TGI. The test agents were microinjected bilaterally in a volume of 100 nl on each side. Drug delivery into the hippocampal CA1 subfield was carried out as previously reported [18, 19]. The animals receiving chloral hydrate anesthesia and surgical preparations without additional experimental manipulations served as sham-controls.

### siRNA administration

All siRNAs were injected into bilateral hippocampal CA1 subfield as previously described [19, 21, 32]. To evaluate transfection efficiency, we used fluorescein isothiocyanate (FITC)-conjugated siRNA as a non-targeting siRNA (sc-36869; Santa Cruz Biotechnology, Santa Cruz, CA, USA). As previously reported, animals were killed 24 h after administration of FITC-siRNA in sham-control and 4 h after TGI/reperfusion before observation under a fluorescence microscope [21]. To inhibit Drp1 expression, we used pre-designed Drp1-siRNA from MISSION® siRNA, (Sigma-Aldrich Ltd.). The sequences were as follows: sense, 5′CAGAGUAUUGUAACACUAU3′, antisense, 5′AUAGUGUUACAAUACUCUG3′. For negative control siRNA (NC), the sequences were as follows: 5′GAUCAUACGUGCGAUCAGA3′, antisense, 5′UCUGAUCGCACGUAUGAUC3′. The final concentration of siRNA was 0.05 nM in a total volume of 400 nl for injection into each side of hippocampal CA1 subfield 24 h before TGI.

### Collection of tissue samples from the hippocampus

At predetermined time intervals (1, 4, 24, or 48 h) after induction of TGI, rats were anesthetized and perfused intracardially with 50 ml of warm (37 °C) saline that contained heparin (100 U/ml). The tissues from bilateral hippocampal CA1 area were collected and concentration of proteins determined as previously reported [19, 21].

### Detection of protein oxidation

The extent of protein oxidation was determined by a commercial kit (OxyBlot, Chemicon, Temecula, CA). Total proteins extracted from the hippocampal CA1 subfield at 24 h after ischemia/reperfusion were subjected to reactions with 2,4-initrophenylhydrazine and derivatized to 2,4-dinitrophenylhydrazone (DNP-hydrazone). Western blotting using a rabbit anti-DNP antibody and then incubated with horseradish peroxidase-conjugated goat anti-rabbit secondary IgG antibody was performed according to manufacturer’s instruction.

### Western blot analysis

Western blot analysis for Drp1 and α-tublin was carried out on proteins extracted from total lysates of hippocampal samples. The primary antibody were Drp1, p-Drp1(Ser616) and active cleaved fragment (17 and 19 kDa) of caspase-3 (Cell Signaling, Danvers, MA, USA), or mouse monoclonal antiserum against α-tubulin (Santa Cruz Biotechnology). The secondary antibody included a horseradish peroxidase-conjugated goat anti-rabbit (Chemicon) for Drp1 and p-Drp1(Ser616), donkey anti-rabbit IgG (Amersham Biosciences, Little Chalfont, U.K.) for activated caspase-3 and goat anti-mouse IgG (Chemicon) for α-tubulin. The specific antibody-antigen complex was detected and measured semiquantitatively as previously reported [19, 21, 32].

### Immunofluorescence staining

Immunofluorescence staining was carried out in animals as reported previously [19]. Briefly, free-floating sections (thickness = 30 μm) of the hippocampus were incubated with a rabbit polyclonal antiserum against p-Drp1 (Ser616) (Cell Signaling) and a mouse monoclonal antiserum, neuron-specific nuclear protein (NeuN, Chemicon). Two secondary antibodies were used that included a goat anti-rabbit IgG-conjugated with Alexa Fluor 488 for p-Drp1(Ser616) and a goat anti-mouse IgG conjugated with Alexa Fluor 568 for NeuN (Molecular Probes, Eugene, OR, USA). The merged images indicated the presence of p-Drp1(Ser616) immunoreactivity in the cytosol and NeuN in the nucleus of neurons. For double immunofluorescence staining of p-Drp1(Ser616) and COXIV, the sections of the hippocampus were first incubated with a rabbit polyclonal antiserum against p-Drp1(Ser616) (Cell Signaling). The sections were subsequently incubated with a goat anti-rabbit IgG conjugated with Alexa Fluor 488 for p-Drp1(Ser616). After fixed with 4 % paraformaldehyde for 5 min, the same sections were incubated with a polyclonal rabbit antiserum against COXIV (Cell Signaling) and then with DyLight 405-conjugated AffiniPure goat anti-rabbit IgG (Jackson ImmunoResearch, West Grove, PA, USA) for labeling COX IV.

### Qualitative and quantitative analysis of DNA fragmentation

Preparations of tissue samples from the hippocampal CA1 subfield for qualitative and quantitative analysis of DNA fragmentation was conducted as reported previously [18, 19]. With total DNA from the hippocampal tissues, nucleosomal DNA ladders were amplified using a DNA ladder assay kit (Maxim Biotech, San Francisco, CA, USA) as previously reported [19, 21]. Samples were separated by electrophoresis on 1 % agarose gels. A cell death enzyme-linked immunosorbent assay (Roche Molecular Biochemicals, Mannheim, Germany) was used to assess the level of histone-associated DNA fragments in the cytoplasm. The amount of nucleosomes in the cytoplasm was determined using 2,20-azino-di-[3-ethylbenzthiazoline] sulfonate as the substrate and the absorbance was measured as previously reported [19, 21].

### Terminal deoxynucleotidyl transferase-mediated dUTP-biotin nick end labeling (TUNEL) staining

Animals were processed for TUNEL staining 48 h after the onset of reperfusion following a 10-min episode of TGI as previously reported [21]. In brief, the hippocampus was removed and fixed in 30 % sucrose in 10 % formaldehyde-saline solution for ≥72 h. Six-micrometer paraffin-embedded sections (thickness = 25 μm) of the hippocampus were processed for TUNEL staining with an apoptosis detection kit (ApopTag, Intergen Company, Purchase, NY, USA). The total numbers of TUNEL-positive cells in each section were counted using an Olympus AX70 microscope and expressed as the TUNEL indices [21].

### Statistical analysis

All values expressed as mean ± SEM. The one-way analysis of variance (ANOVA) was used, as appropriate, to assess group means, followed by the Scheffe’ multiple-range test for post-hoc assessment of individual mean. *P* < 0.05 indicates statistical significance.

## Results

### Tem7poral changes of drp1 expressions in the hippocampal CA1 subfield after TGI

**Fig. 1:**
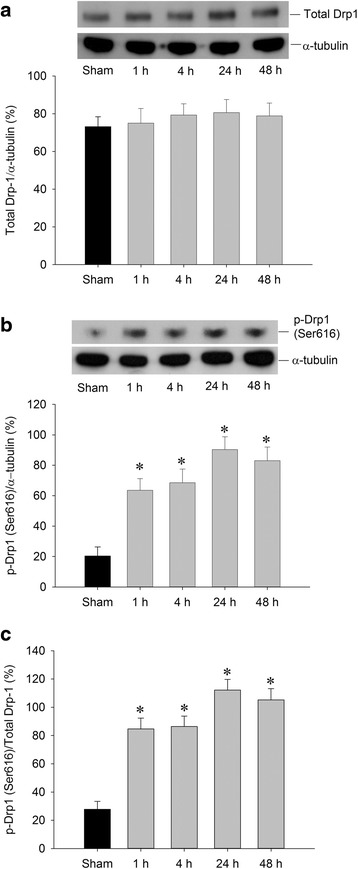
Transient induction of p-Drp1(Ser616) by TGI. Hippocampal CA1 samples were collected from the rats at indicated times after 10-min TGI or sham-operated controls followed by protein extraction and western analysis for detection of total Drp1 in (**a**) and p-Drp1(Ser616) in (**b**). The same blots were also probed with α-tubulin antibody to serve as an internal reference control for equal loading of proteins in each lane. The ratio change of p-Drp1(Ser616)/total Drp was shown in (**c**). Values are mean ± SEM from representative blots and quantitative analyses from 4-6 animals in each experimental group are shown. *P < 0.05 versus sham control group in the Scheff′e multiple-range test

We first examined whether Drp1 is induced by TGI in the hippocampal CA1 subfield. The division of the mitochondria, which is required for apoptosis as well as normal cell growth and development, is controlled in part by the phosphorylation of Drp1 at Ser616 by Cdk1/cyclin B [33, 34]. We used Drp1 and phosphorylation of Drp1 at Ser616 (p-Drp1(Ser616)) antibodies to examine the expression in the hippocampal CA1 subfield after TGI at the designed time. Total Drp1 (Fig. 1a) revealed no significant change and p-Drp1(Ser616) significantly increased as early as 1 h after TGI and remained elevated until 24 and 48 h after TGI in the total protein extracted from the hippocampal CA1 subfield (Fig. 1b, c). This finding may suggest the active form, phosphorylated Drp-1(Ser616), in stead of total Drp1 expression, plays the functional role on the fission process of mitochondria in this ischemic condition. This notion was reported before that Drp1 phosphorylation at serine 616 results in its activation and recruitment to mitochondria [35].

### Effect of Drp1 inhibitor over p-Drp1(Ser616) expression, oxidative stress and neuronal injury in the hippocampal CA1 subfield After TGI

**Fig. 2:**
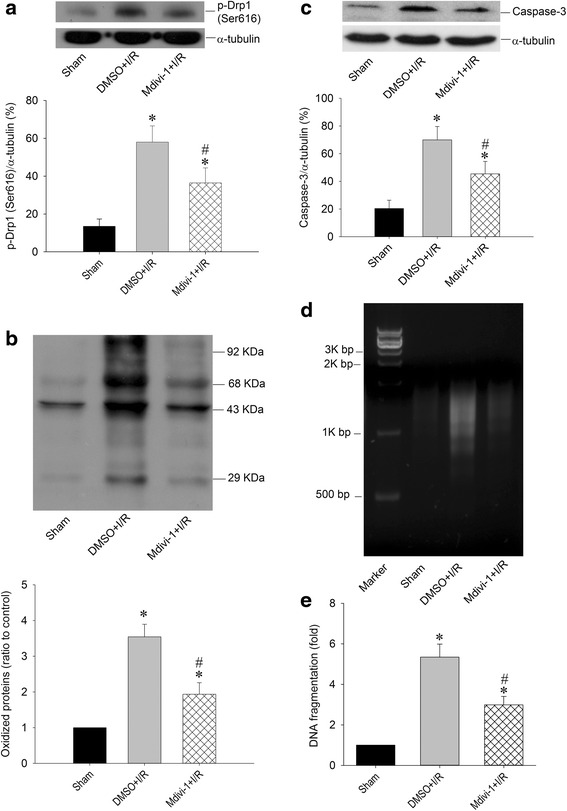
Mdivi-1 reduced Drp1 phosphorylation, protein oxidation, and DNA fragmentation after TGI. Rats were treated intraperitoneally with Drp1 inhibitor Mdivi-1 (2.4 mg/kg) or its solvent DMSO 30 min before TGI. Total proteins were extracted from the hippocampal CA1 subfield of sham-operated controls or treated animals 24 h after 10-min TGI for detection of p-Drp1(Ser616) in (**a**), protein oxidation in (**b**) and activated caspase-3 expression in (**c**). DNA was isolated from the hippocampal CA1 subfield of sham-operated controls, DMSO + I/R and Mdivi-1 + I/R 48 h after TGI for detection of DNA fragmentation by PCR assay (**d**), protein lysates from hippocampal CA1 tissues were collected 48 h after TGI for detection of DNA fragmentation by sandwich ELISA in (**e**). Values are mean ± SEM from representative blots and quantitative analysis from 5–6 animals in each experimental group (**a**, **b** and **c**). Values in (**e**) are fold changes with reference to sham-operated controls and are mean ± SEM of 4 animals in each experimental group. *P < 0.05 versus sham control group, #*P* < 0.05 versus DMSO + I/R group in the Scheffé multiple-range test. I/R: ischemia/reperfusion

Mitochondrial biogenesis may function as an endogenous protective mechanism [19, 20] while mitochondrial fission may initiate the apoptotic process under ischemic insult [7, 8]. As Drp1 phosphorylation at Ser616 in rat hippocampal CA1 regions shows a significant change that peaked at 24 h after TGI, with no significant change in total Drp1 protein expression after 1–48 h of reperfusion, we then test if downregulation of p-Drp1(Ser616) expression will exert beneficial effect over hippocampal CA1 subfield under TGI. Firstly, we tested the effects of Mdivi-1, a selective inhibitor of Drp1, over TGI-induced p-Drp1(Ser616) expression. Western blot analysis revealed a reduction of p-Drp1(Ser616) protein level in the hippocampal CA1 subfield 24 h after TGI in the Mdivi-1-treated group as compared to the vehicle groups (Fig. 1a). An excessive production of ROS underlies neuronal cell death in the CA1 subfield of the hippocampus after TGI [18, 19]. We therefore investigated the effects of Mdivi-1, which is capable of reducing TGI-induced phosphorylation of Drp1 at Ser616 (Fig. 2a), over ischemia-dependent oxidative stress and apoptosis related molecule. We found that, at 24 h following TGI, protein oxidation as well as activated caspase-3 expression in the hippocampal CA1 subfield decreased significantly by pretreatment with Mdivi-1 (Fig. 2b, c). We have also shown that an excessive production of ROS underlies DNA fragmentation and neuronal damage in the hippocampal CA1 subfield after TGI [18, 19]. Our results derived from both qualitative (Fig. 2d) and quantitative (Fig. 2e) analyses of DNA fragmentation indicated that, at 48 h following TGI, apoptosis-like cell death decreased significantly by pretreatment of Mdivi-1. Thus, Mdivi-1 capable of reducing the extent of phosphorylation Drp1 at Ser616 also attenuates the TGI-mediated oxidative damage and neuronal injury in rat hippocampal CA1 regions.

### Drp1-siRNA silences Drp1 expression, attenuates oxidative stress and lessens neuronal Injury in the hippocampal CA1 subfield after TGI

**Fig. 3:**
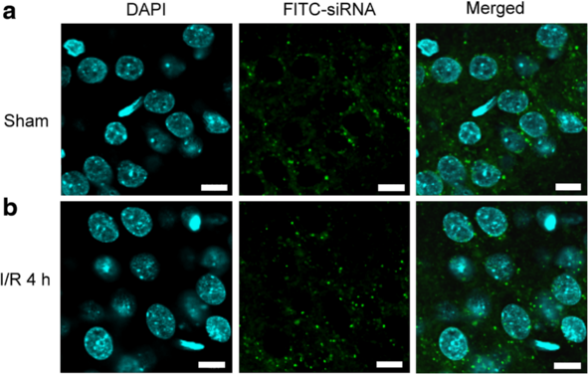
Successful delivery of siRNA into hippocampal CA1 subfield. Fluorescent double staining of FITC-siRNA (green) and DAPI (blue) were observed in the hippocampal CA1 subfield 24 h after injection of 400 nl FITC-siRNA (10 μM), which was distributed in the cytosol of hippocampal CA1 in both sham-control (**a**) and in the rats subjected to TGI-reperfusion for 4 h (**b**). Scale bar: 10 μm. I/R: ischemia/reperfusion

**Fig. 4:**
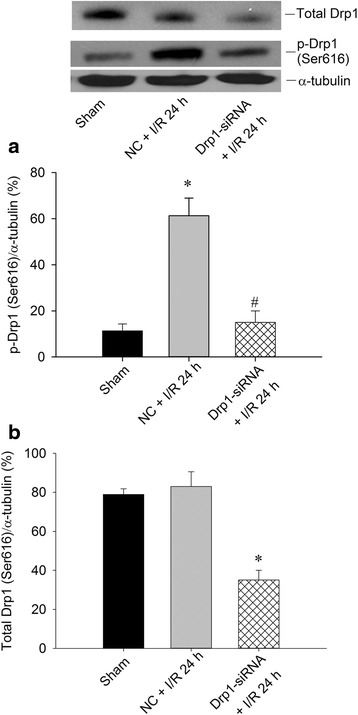
Western blotting of p-Drp1(Ser616) and total Drp1 expression after Drp1-siRNA in the hippocampal CA1 subfield after TGI. After microinjection with Drp1-siRNA (0.05 nM in a total volume of 400 nl) into the CA1 subfield 24 h before TGI, total proteins were isolated from hippocampal CA1 subfield of sham-operated controls, control siRNA with TGI, or Drp1-siRNA animals after 10 min of TGI with 24 h reperfusion for detection of p-Drp1(Ser616) in (**a**) and total Drp1 in (**b**). The same blots were also probed with a α-tubulin antibody to serve as an internal control for equal loading of proteins in each lane. Values are mean ± SEM from representative blots and quantitative analysis from 4-6 animals in each experimental group. I/R: ischemia/reperfusion, NC: negative control siRNA

**Fig. 5:**
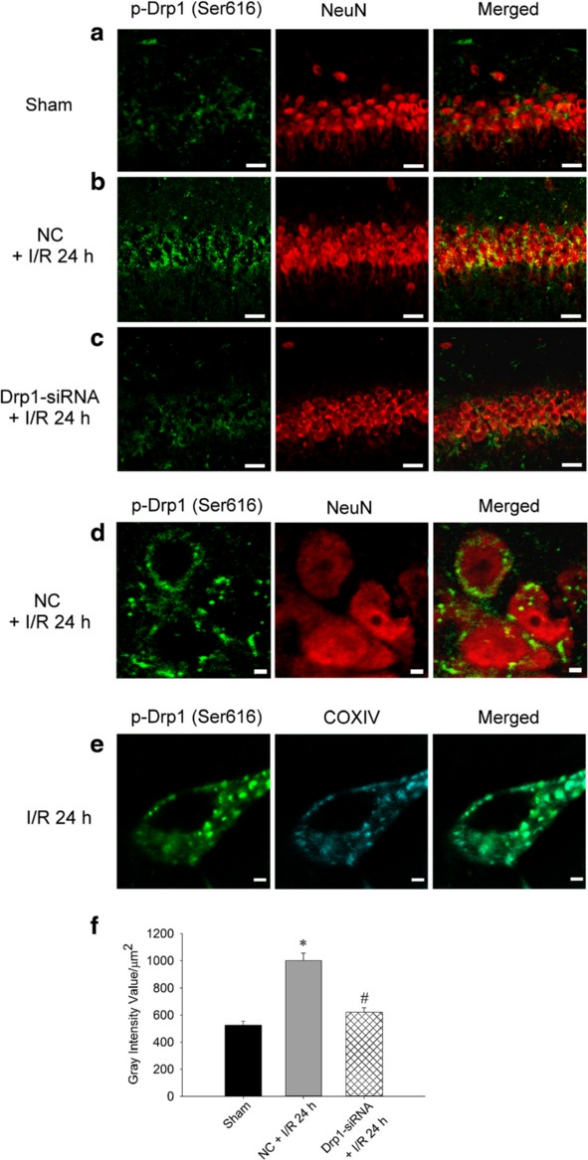
Drp1-siRNA downregulates p-Drp1(Ser616) expression in the hippocampal CA1 subfield after TGI. Fluorescent double staining of p-Drp1 (green) and NeuN (red) in the hippocampal CA1 subfield in **a** sham control group, **b** ischemia/reperfusion 24 h with negative control siRNA and **c** siRNA for Drp1 and ischemia/reperfusion 24 h. NeuN showed the nuclear distribution while p-Drp1 were dispersed in the cytoplasm. Scale bars, 50 μm Merged images with higher magnification demonstrate that p-Drp1(Ser616) and NeuN-positive cells localized separately in the nucleus and non-nuclear cytoplasm in neurons in (**d**). Scale bars, 2 μm. A semi-quantitative data about the change of p-Drp1(Ser616) expression after Drp1-siRNA for Fig. 5 a-c was shown in (**f**). Fluorescent double staining of p-Drp1(Ser616) (green) and COXIV (blue) in the neuron of the hippocampal CA1 subfield; merged image shows the co-localization in mitochondria in neurons under the condition of ischemia/reperfusion for 24 h (**e**). Scale bars, 2 μm. I/R: ischemia/reperfusion, NC: negative control siRNA. COXIV: cytochrome c oxidase subunit 4

**Fig. 6:**
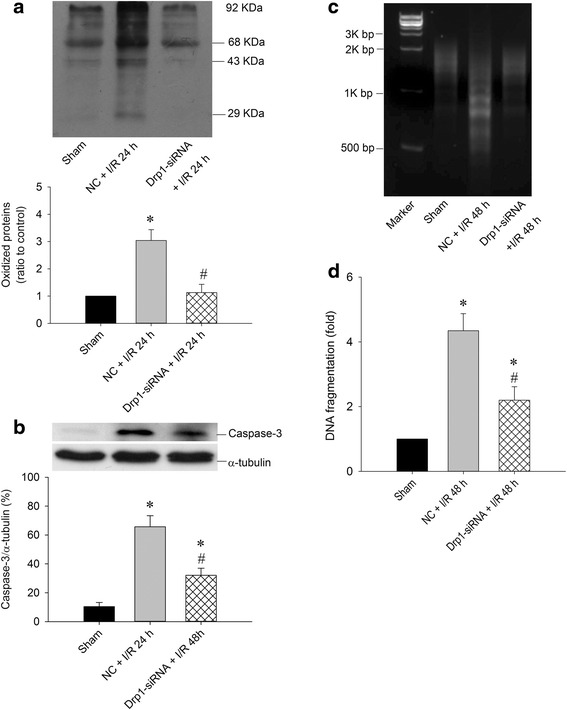
Drp1 siRNA attenuates oxidative stress and decreases DNA fragmentation in hippocampal CA1 subfield after TGI. After microinjection with Drp1 siRNA (0.05 nM in a total volume of 400 nl) into the CA1 subfield 24 h before TGI, Total proteins were isolated from the hippocampal CA1 subfield of sham-operated controls, control siRNA with TGI, or Drp1 siRNA with TGI for protein oxidation in (**a**) and activated caspase-3 expression in (**b**). DNA was isolated from collected hippocampal CA1 subfield of sham-operated controls, vehicle with negative control siRNA, and Drp1-siRNA 48 h after TGI for detection of DNA fragmentation by PCR assay in (**c**) Hippocampal CA1 tissues were collected 48 h after TGI for detection of DNA fragmentation by sandwich ELISA in (**d**). Values are mean ± SEM from representative blots and quantitative analysis from 5–6 animals in each experimental group (**a** and **b**). Values are fold changes in (**d**) with reference to sham-control; mean ± SEM of 5–7 animals in each experimental group. *P < 0.05 vs. sham-control group and #*P* < 0.05 vs. negative control siRNA + I/R in the Scheff′e multiple-range test. I/R: ischemia/reperfusion, NC: negative control siRNA

To further clarify the pivotal role of Drp1 in this ischemic paradigm of the brain, we also employed molecular approach by using Drp1-siRNA to knock down its expression. We first confirmed its efficient delivery by injecting FITC-conjugated non-targeting siRNA (FITC-siRNA) into the hippocampal CA1 subfields bilaterally. Immunofluorescence demonstrated the cytosolic distribution of FITC-siRNA, in both the sham (Fig. 3a) and TGI (Fig. 3b) groups, in the hippocampal CA1 subfield 24 h after injection. These results indicated the siRNA could be successfully delivered into neurons under both sham-operated and ischemia/reperfusion condition. We then silenced the Drp1 protein expression by this approach. We found that Drp1-siRNA decreased p-Drp1(Ser616) protein level in hippocampal CA1 subfield 24 h after TGI (Fig. 4a). Again as expected, total Drp1 revealed no significant change between sham and negative control (NC) siRNA + TGI group but showed decreased expression in Drp1-siRNA + TGI group (Fig. 4b). In parallel with the findings from Western blotting, under a laser-scanning confocal microscope, more p-Drp1(Ser616)-positive neurons, as revealed by its colocalization to the NeuN-positive cells, were observed in the hippocampal CA1 subfield at the same time point (Fig. 5b) as compared to the sham control animals (Fig. 5a). Pictures of higher magnification were shown in 5D. Microinjection of Drp1-siRNA into the hippocampus markedly reduced the numbers of p-Drp1(Ser616)-positive neurons induced by TGI under the same experimental conditions (Fig. 5c, f). Fluorescent double immunostaining of p-Drp1(Ser616) and COXIV at higher magnification revealed co-localization of these two proteins in the mitochondria of hippocampal CA1 neurons at 24 h following TGI (Fig. 5e). Pretreatment with Drp1-siRNA significantly retarded the extent of protein oxidation and activated caspase-3 expression in the hippocampal CA1 subfield 24 h after TGI (Fig. 6a, b). Both qualitative (Fig. 6c) and quantitative (Fig. 6d) analyses revealed that downregulation of p-Drp1(Ser616) by siRNA significantly attenuated TGI-induced DNA fragmentation in the CA1 subfield of rat hippocampus. These findings illustrated that inhibition of p-Drp1(Ser616) expression using Drp1-siRNA may decrease oxidative neuronal damage and DNA fragmentation in the hippocampal CA1 subfield after TGI.

### Effect of PPARγ agonist on p-Drp1(Ser616) expression, oxidative stress and neuronal injury in the hippocampal CA1 subfield after TGI

**Fig. 7:**
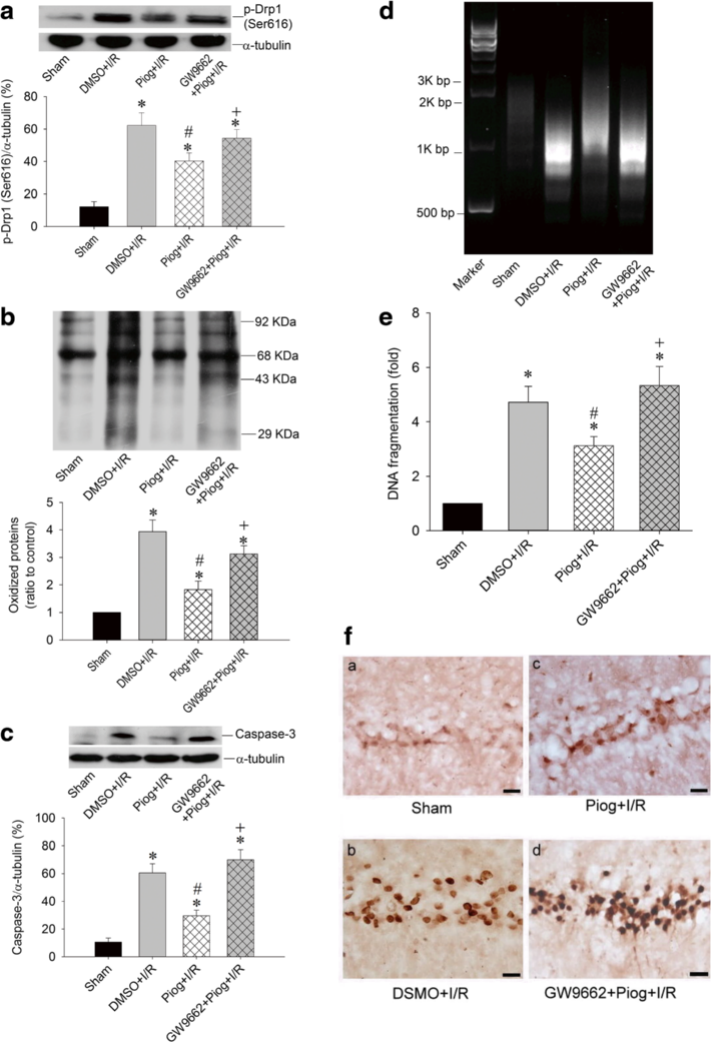
Pioglitazone regulates Drp1 phosphorylation, protein oxidation, DNA fragmentation, and neuronal apoptosis in a PPARγ-dependant pathway after TGI. The chemical compounds microinjected into bilateral CA1 subfields as following with DMSO, pioglitazone (20 nmol) 30 min before TGI, or GW9663 (500 ng) 30 min before pioglitazone and 60 min before TGI. Total proteins were isolated from the hippocampal CA1 subfield of sham-operated controls or treated animals 24 h after 10 min of TGI for detection of p-Drp1 (Ser616) in (**a**) and protein oxidation in (**b**) and activated caspase-3 expression in (**c**). DNA was isolated from collected hippocampal CA1 subfield of sham-operated controls, DMSO + I/R, pioglitazone + I/R and GW9662 + pioglitazone 48 h after TGI for detection of DNA fragmentation by PCR assay (**d**) and hippocampal CA1 tissues were collected 48 h after TGI for detection of DNA fragmentation by sandwich ELISA in (**e**). Hippocampal slices were subjected to TUNEL staining to determine the extents of apoptosis in (**f**) which showed sham control in (*a*), ischemia/reperfusion with vehicle control in (*b*), pioglitazone with ischemia/reperfusion in (*c*) and GW9662 + pioglitazone and ischemia/reperfusion in (*d*). Values are mean ± SEM from representative blots and quantitative analyses from 5–6 animals in each experimental group (**a**, **b** and **c**); values in (**e**) are fold changes with reference to sham-control; mean ± SEM of 5-6 animals in each experimental group. *P < 0.05 vs. sham-control group, #*P* < 0.05 vs. DMSO + I/R and + *P* < 0.05 versus Piog + I/R group in the Scheff′e multiple-range test. I/R: ischemia/reperfusion. Piog: pioglitazone

Although PPARγ agonist including rosiglitazone or pioglitazone can reduce inflammation and oxidative damage as well as diminishes cell death caused by ischemic injury [23–27], whether PPARγ-dependent pathway can reduce the expression of p-Drp1(Ser616) and ameliorate hippocampal injury induced by global ischemia is currently unknown. To address this issue, pioglitazone (20 nM) was microinjected into the CA1 subfield 30 min before TGI with or without prior microinjection of GW9662, a PPARγ antagonist, (500 ng) 30 min before pioglitazone. Western blotting revealed an increased p-Drp1(Ser616) protein level in hippocampal CA1 subfield 24 h after TGI, which was reduced by pioglitazone pretreatment; moreover, GW9662 reversed the pioglitazone effect over p-Drp1(Ser616) protein expression (Fig. 7a). In parallel with the p-Drp1(Ser616) protein expression, pretreatment with pioglitazone decreased TGI-induced protein oxidation and activated caspase-3 expression, whereas GW9662 partially reversed this beneficial effect of pioglitazone (Fig. 7b, c). Both qualitative (Fig. 7d) and quantitative (Fig. 7e) studies of DNA fragmentation revealed that pioglitazone in part lessened TGI-induced DNA fragmentation, which was reversed by GW9662 pretreatment. Consistently, the extent of hippocampal neuronal apoptosis based on TUNEL staining, revealed the same tendency after pioglitazone and GW9662 treatments (Fig. 7f).

## Discussion

The results demonstrate that TGI increases p-Drp1(Ser616) expression, a phosphorylation site important for increasing mitochondrial fission, in the hippocampal CA1 subfield after TGI. In contrast, no significant change for total Drp1 expression under TGI. Both the Drp1 inhibitor Mdivi-1 and the siRNA targeting Drp1 decreased p-Drp1(Ser616) expression, lessened protein oxidation, and attenuated neuronal damage in the hippocampal CA1 subfield. These findings suggested the pivotal role of p-Drp1(Ser616) in TGI-induced neuronal injury. PPARγ agonist, pioglitazone, reduced the p-Drp1(Ser616) expression, decreased TGI-induced oxidative stress, lessened the extents of DNA fragmentation, and diminished the numbers of TUNEL-positive neuronal cells; all of these effects were reversed by GW9662, a PPARγ antagonist. These findings indicated that PPARγ-dependent pathway can reduce the expression of p-Drp1(Ser616) and ameliorate hippocampal injury induced by global ischemia.

Mitochondrial fission that occurs as an early event of neuronal cell death plays a pivotal role in cerebral ischemia [7]. Drp1 is a large GTPase that cycles between the cytosol and mitochondrial outer membrane to function as a key contributor in the mitochondrial dynamic process when the cells encounter various stressful stimuli, whereas Drp1-mediated mitochondrial fission and downstream mitochondrial death pathways are critically involved in the observed cell death [7, 36]. Phosphorylation of Drp1 is crucial to regulating mitochondrial dynamics [37]. Multiple phosphorylation sites have been characterized for their functional importance [34]. Drp1 phosphorylation at serine 616 can result in its activation and recruitment to mitochondria [35]. On the contrary, fission is inhibited when Drp1 is phosphorylated at Ser637 [34, 38]. The role of Drp1 in cerebral ischemia is just beginning to emerge [8, 9, 39]. It has been well demonstrated that knockdown of the fission protein Drp1 or with Drp1 inhibitors can block toxicity in a glutamate-induced oxidative stress model in HT22 cells and Drp1 inhibitors-Mdivi a or Mdivi b can reduce infarct volume in a mouse model of transient focal ischemia [8]. However, the protective mechanism involving inhibition of Drp1 or Drp1 phosphorylation is still awaited to clarify with in vivo cerebral ischemia study. After focal cerebral ischemia, Drp1 phosphorylation is related to the apoptotic process in peri-infarct regions [39]. In our recent study, cerebral ischemia increased p-Drp1(Ser616) expression with no evident change over total Drp1 and p-Drp1(Ser637) expression; further, down-regulation of PINK1 increased p-Drp1(Ser616) expression, heightened DNA oxidation, and augmented neuronal damage in the hippocampal CA1 subfield [21]. These studies denoted a pivotal role of p-Drp1 in cerebral ischemia and attenuation of p-Drp1 (Ser616) levels may exert neuroprotective effects. In a recent report, preventing dephosphorylation of Drp1(Ser637) with Mdivi-1 or Drp1-siRNA can preserve mitochondrial networking and ultrastructure following heart ischemia/reperfusion model [40].

We have reported before that TGI-induced ROS generation results in heightened protein oxidation and neuronal death in the hippocampal CA1 subfield [18, 19]. In this work, concomitantly with heightened protein oxidation, p-Drp1(Ser616) expression was increased (Fig. 2). It was known that Drp1 mediates mitochondrial fission [41, 42]. Drp1 activity results from phosphorylation by cyclin B/cyclin-dependent kinase (CDK), which causes phosphorylation of Serine 616 and promotes Drp1 recruitment to mitochondria for subsequent fission [41, 42]. The roles of calcium cascade in the cerebral ischemic paradigm in terms of necrosis and apoptosis are well established [43–45]. It was reported that calcium influx across the plasma membrane was an upstream event governing mitochondrial fission and ROS generation that can be reversed by calcium chelation [46]. Increased intracellular calcium may cause Drp1 activation in cardiac ischemia [40]. The identification of mitochondrial calcium uniporter holds important clinical perspective, which allows the rapid calcium accumulation across the inner mitochondrial membrane [47]. It was demonstrated that under ischemia/reperfusion injury, mitochondria accumulate significant amounts of calcium from the cytosol via mitochondrial calcium uniporter and blocking mitochondrial calcium uniporter was demonstrated to exert protective effects against ischemia/reperfusion injury [48]. In a recent study, it revealed that mitochondrial calcium uniporter regulates the process of mitochondrial fission by controlling the calcium transport, directly upregulating mitochondrial fission proteins Drp1 [49]. All these evidence denote the importance of calcium and mitochondrial calcium uniporter in mitochondrial dynamics under ischemic condition.

It was suggested that mitochondrial oxidative stress modulates Drp1 expression and causes an imbalance between mitochondrial fission and fusion, resulting in mitochondrial fragmentation and thus contributing ultimately to cellular dysfunction [50]. Treatment of antioxidants such as vitamin E or MitoQ can lessen mitochondrial fragmentation and Drp1 expression [51, 52]. On the contrary, it was shown that knockdown of Drp1 expression suppressed production of mitochondrial ROS [53]. Both inhibition of Drp1 expression with antisense oligonucleotide and a dominant-negative mutant of Drp1 decrease oxidative stress [54, 55]. In this study, we showed that Mdivi-1 decreased p-Drp1(Ser616) expression, lessened protein oxidation as well as activated caspases 3, a marker of oxidative stress and apoptosis respectively (Fig. 2b, c). Mdivi-1 affects total Drp1 expression as well as phosphorylation level though the underlying mechanism is not well understood [33, 56]. Mdivi-1 attenuates mitochondrial division by blocking dynamin GTPase activity, impedes apoptosis by inhibiting mitochondrial outer membrane permeabilization and effectively hinders Bid-activated Bax/Bak-dependent cytochrome *c* release from mitochondria [57].

We then used siRNA strategy to confirm the crucial role of Drp1 in this ischemic paradigm. Based on immunofluorescence studies, we verified the successful delivery of siRNA and reducing p-Drp1(Ser616) expression (Figs. 3, 4 and 5). In supporting the regulatory role of Drp1 over oxidative stress in ischemic condition, Drp1-siRNA treatment decreased p-Drp1(Ser616) expression (Figs. 4 and 5), which was accompanied by attenuated protein oxidation, lessened apoptotic process and decreased neuronal damage (Fig. 6). Based on these results, Drp1-dependent enhancement of ROS generation is a more favorable assumption than oxidative stress-mediated induction of Drp1, at least in our TGI paradigm. This observation may be vital for therapeutic purposes for ischemic stroke by impeding the activation of p-Drp1(Ser616) expression rather than inhibition of oxidative stress only.

In this study, we found that PPARγ agonist- pioglitazone decreased p-Drp1(Ser616) expression, lessened oxidative stress as well as apoptotic process, and attenuated neuronal damage under TGI/reperfusion in the hippocampal CA1 subfield, all of which were reversed by GW9662 (Fig. 7). PPARγ is a ligand-activated transcriptional factor that belongs to the nuclear hormone receptor superfamily and influences the expression of genes under various physiological or pathological conditions, including redox balance and vascular integrity [58]. We and others have shown before that thiazolidinediones drugs, including rosiglitazone and pioglitazone, possess characters of PPARγ agonist exerting neuroprotective effects in various models, which were abrogated by GW9662, a PPARγ antagonist [31, 59, 60]. Several potential mechanisms related to PPARγ and mitochondrial dynamic were reported before. Pioglitazone demonstrates the ability to restoring mitochondrial defects and mitochondrial networking [61]. Another PPARγ agonist, ciglitazone, can prevents mitochondrial size reduction in hippocampal neurons induced by H_2_O_2_ [62]. The potential mechanism may relate to the property of PPARγ being able to directly induce the expression of several mitochondria-related proteins including Drp1. PPARγ agonists have been linked to calcium balance [63, 64], which may affect Drp1 phosphorylation [65] and offer the ability of PPARγ to control mitochondrial dynamics modulation. Another common point for the mitochondrial dynamics modulation among these PPARγ agonist is the capability of activating PPARγ coactivator 1-α [66, 67], an important molecule that can counteract excessive oxidative stress and apoptosis in cerebral ischemia [68]. Thus, pioglitazone has the potential to reduce neuronal injury after cerebral ischemia through mitochondrial dynamic-related proteins and PPARγ-dependent pathways.

## Conclusion

In summary, the present study demonstrated that inhibition of TGI-induced p-Drp1(Ser616) expression by Drp1 inhibitor and Drp1-siRNA can decrease protein oxidation, apoptotic process and neuronal damage in the hippocampal CA1 subfield. PPARγ-dependent pathway can decrease the expression of p-Drp1(Ser616) and protect hippocampal CA1 injury induced by global ischemia.

## Abbreviations

ANOVA: one-way analysis of variance
CDK: cyclin-dependent kinase
COXIV: cytochrome c oxidase subunit 4
Drp1: dynamin-related protein 1
FITC: fluorescein isothiocyanate
p-Drp1(Ser616): phosphorylation at Drp1serine 616
PPARγ: peroxisome proliferator-activated receptor-gamma
TGI: transient global ischemia
TUNEL: terminal deoxynucleotidyl transferase-mediated dUTP-biotin nick end labeling
